# First-trimester inflammation and dyslipidemia in preterm delivery: the role of monocyte-to-HDL cholesterol ratio and lipid profiles

**DOI:** 10.1007/s11845-025-03992-7

**Published:** 2025-06-23

**Authors:** Didem Kaymak, Simge Berrak Beyoglu Oruc, Ebru Alıcı Davutoğlu

**Affiliations:** 1 Department of Obstetric and Gynecology, Division of Perinatology, Istanbul Education and Research Hospital, Istanbul, Turkey; 2 Department of Obstetric and Gynecology, Bahcesehir University School of Medicine, Medical Park Goztepe Hospital, Istanbul, Turkey; 3 Department of Obstetric and Gynecology, Division of Perinatology, Istanbul University-Cerrahpasa, Cerrahpasa School of Medicine, Istanbul, Turkey

**Keywords:** First-trimester biomarkers, Inflammation, Maternal lipid profile, Monocyte-to-HDL ratio (MHR), Preterm delivery

## Abstract

**Background:**

Preterm delivery (PTD) remains a leading cause of neonatal morbidity and mortality. Maternal inflammatory disturbances during early pregnancy may contribute to PTD pathogenesis. Alterations in lipid metabolism, particularly decreased high-density lipoprotein cholesterol (HDLc) and the HDLc-to-low-density lipoprotein cholesterol (HDLc/LDLc) ratio, alongside inflammatory markers such as the monocyte-to-HDLc ratio (MHR), have been associated with adverse pregnancy outcomes. However, their predictive role for PTD requires further investigation.

**Aim:**

This study aimed to evaluate the relationship between first-trimester maternal lipid profiles, HDLc, HDLc/LDLc ratio, and MHR, and the subsequent risk of PTD.

**Methods:**

A retrospective analysis was conducted on 152 pregnant women, including 53 PTD cases and 99 term deliveries. First-trimester complete blood counts and lipid profiles were assessed. MHR was calculated as the monocyte count divided by the HDLc level. Regression and correlation analyses evaluated associations, while receiver operating characteristic (ROC) curve analysis assessed MHR’s predictive value for PTD.

**Results:**

PTD cases exhibited significantly lower HDLc levels and HDLc/LDLc ratios and higher MHR values (*p* < 0.05). HDLc showed a negative correlation with PTD risk (*r* =  − 0.308, *p* = 0.000), while MHR correlated positively (*r* = 0.250, *p* = 0.002). ROC analysis identified an MHR cut-off of 0.0078 for PTD prediction (AUC = 0.652, 95% CI 0.562–0.741, *p* = 0.002).

**Conclusion:**

First-trimester elevated MHR and reduced HDLc levels may serve as early biomarkers for PTD risk, reflecting underlying inflammatory and metabolic dysregulation. Early assessment of MHR could enhance risk stratification and guide preventive strategies. Further studies are warranted to validate these findings.

## Introduction

Preterm delivery (PTD), characterized as delivery before 37 weeks of gestation, contributes to approximately 69% of perinatal mortality and a significant portion of long-term morbidity [1, 2]. Indeed, there has been an escalation in PTD rates in high-income countries during the past two decades. Factors contributing to this include a rise in multiple pregnancies, a greater incidence of obstetric interventions, and an elevated average mother age [3]. The etiology of PTD remains unclear; however, factors including maternal vascular issues, infections, and inflammation have been proposed as possible contributors [4].

Circulating monocytes exhibit tissue factor expression in inflammatory or pro-thrombotic conditions, transitioning to a procoagulant phenotype. High-density lipoprotein cholesterol (HDLc) inhibits monocyte tissue factor expression by blocking p38 activation and phosphoinositide 3-kinase. It mitigates monocyte-induced inflammation and oxidation by preventing macrophage migration and promoting cholesterol efflux. Additionally, HDLc protects endothelial cells from inflammation and oxidative stress by modulating monocyte activity and precursor proliferation [5, 6]. Elevated lipid levels play a role in providing hormonal and nutritional support during a healthy pregnancy; however, excessively high levels may lead to oxidative stress and have been associated with adverse birth outcomes in animal studies and atherosclerosis in human offspring [7–9]. Research indicates that the incidence of PTD increases in pregnant women with elevated triglycerides (TG) and non-HDLc, whereas it decreases in those with high HDLc levels [10–12].

The monocyte count to HDL cholesterol ratio (MHR) was first defined by Kanbay et al. in 2014, with findings indicating that elevated MHR values may predict the risk of adverse cardiovascular outcomes [13]. A high MHR value, along with elevated monocyte levels and low HDLc levels, may signify increased inflammation [13]. MHR is linked to various conditions, such as hypertension, chronic kidney disease, abdominal aneurysm, intracerebral hemorrhage, gestational diabetes mellitus, and metabolic syndrome in individuals with polycystic ovary syndrome [14–16]. This study aimed to examine the association between serum lipid levels and calculated MHR during the first trimester of pregnancy and the incidence of preterm birth.

## Materials and method

This research involved 152 pregnant women, consisting of 53 with PTD and 99 with term birth, as well as gestation-matched healthy controls, all diagnosed and treated at the Agri Education and Research Hospital from January 2022 to April 2023. Approval from the ethics committee was obtained, and a retrospective review of the patients’ files was conducted.

Gestational age was determined based on the last menstrual period, and for cases with uncertainty, it was assessed using the first trimester crown-rump length measurement. PTD was characterized as deliveries occurring before 37 weeks of gestation. Spontaneous preterm labor was characterized by intact membranes, regular contractions, and cervical changes (≥ 2 cm dilation) occurring without labor induction. Preterm premature rupture of membranes (PROM) is defined as the rupture of membranes occurring either before or simultaneously with the start of spontaneous contractions. Women undergoing either spontaneous labor or PROM resulting in preterm delivery were put together as a single spontaneous preterm delivery group for analysis. In our clinical setting, first-trimester lipid panels and complete blood counts are routinely requested by family physicians in primary care as part of standard antenatal follow-up. Therefore, blood samples used in this study were originally obtained for routine clinical purposes. All laboratory measurements were performed between 8 and 14 weeks of gestation, based on the time of initial antenatal presentation.

Women who delivered during the designated timeframe and possessed complete blood count, HDLc, low-density lipoprotein cholesterol (LDLc), total cholesterol (TC), and TG data from the first trimester of pregnancy were included in the study. Monocyte counts were derived from CBC results, which were performed using an automated hematology analyzer. Patients with gestational diabetes mellitus, preeclampsia, systemic diseases, and acute or chronic inflammatory diseases, including multiple gestations, hematopoietic system disorders, and malignancies, as well as those lacking any of the blood test parameters, were excluded. The maternal age, gravidity, parity, and history of abortus, birth week, and birth weight were recorded from medical records. Monocyte, HDLc, LDLc, TC, and TG levels were documented. The MHR was determined by dividing the number of monocytes by the HDLc count, whereas the ratios of HDLc to LDLc (HDLc/LDLc), total cholesterol (HDLc/TC), and triglycerides (HDLc/TG) were calculated from cholesterol levels. To enhance statistical power, a 2:1 control-to-case ratio was used. Term controls were randomly selected from women who delivered at term during the same period and who also had available first-trimester laboratory results. No direct matching was applied; however, controls were selected within the same delivery window to ensure comparable follow-up and laboratory timing. A post hoc power analysis was conducted to evaluate sample adequacy. Assuming a medium effect size (Cohen’s *d* = 0.5), a total of 53 preterm and 99 term cases provided a statistical power of approximately 83.6% at a significance level of 0.05, indicating that the study was sufficiently powered to detect meaningful differences between the groups.

All analyses were conducted utilizing version 26.0 of the Statistical Package for the Social Sciences (SPSS) software (Chicago, IL, USA). The Kolmogorov–Smirnov test was employed to evaluate the normality of variable distributions. Data were presented as mean ± standard deviation. Categorical variables were represented as frequencies and/or percentages. An ANOVA test or the Kruskal–Wallis test was used to compare continuous numeric variables, regardless of their distribution normality. Pearson correlation analysis was applied to assess the relationships between normally distributed variables, including HDLc and the HDLc/TC ratio, with preterm delivery status, birth weight, and gestational age. For non-normally distributed parameters, including MHR, HDLc/LDLc, and HDLc/TG ratios, Spearman rank correlation analysis was performed to evaluate their associations with preterm delivery, birth weight, and gestational age. The optimal cut-off values of MHR for distinguishing between patients and control groups were assessed by ROC analysis, calculating AUC to achieve the highest sum of sensitivity and specificity for the significant test. A *p*-value less than 0.05 was deemed statistically significant.

## Results

**Fig. 1:**
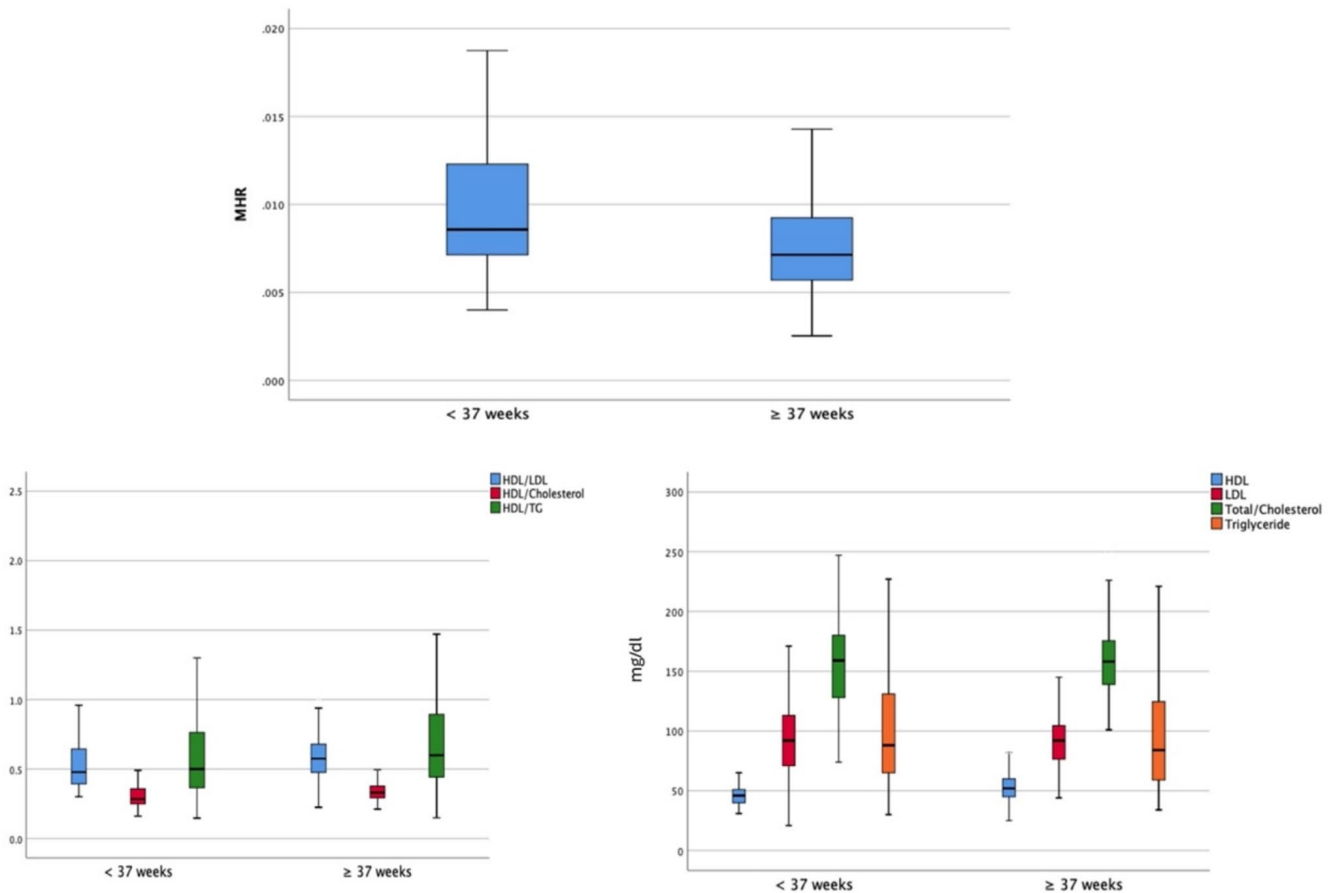
The levels of monocyte, HDLc, LDLc, total cholesterol, triglyceride and the MHR, HDLc/LDL, HDLc/total cholesterol, and HDLc/triglyceride of patients. MHR: monocyte-to-high-density lipoprotein ratio, HDLc: high-density lipoprotein cholesterol, LDLc: low-density lipoprotein cholesterol, MHR: monocyte-to-HDLc ratio

Table 1 presents the demographic and clinical characteristics of all patients. Birth week and birth weight were higher in the term group (*p* < 0.005). The mean gravidity, parity, and frequency of abortus history did not differ between the groups. The levels of monocyte, HDLc, LDLc, TC, and TG and the MHR, HDLc/LDLc, HDLc/TC, and HDLc/TG are shown in Table 2 and Fig. 1. The PTD group displayed significantly higher MHR and significantly lower HDLc and HDLc/LDLc compared to the group with term delivery.

**Table 1:** Baseline demographic and clinical characteristics of the study population

	Preterm group *N:*53	Term group *N:*99	*p*
Age (years)	27.5 ± 5.9	28.5 ± 5.2	0.176
Gravida	2.9 ± 2	3.2 ± 1.8	0.425
Parity	1.5 ± 1.6	1.7 ± 1.5	0.307
History of abortus	28.3 (15/53)	36.3 (36/99)	0.370
Birth week	34 ± 2.7	38.9 ± 1.2	** < 0.001**
Birth weight (g)	2363 ± 571	3214 ± 341	** < 0.001**

**Table 2:** The levels of monocyte, HDLc, LDLc, total cholesterol, and triglyceride and the MHR, HDLc/LDL, HDLc/total cholesterol, and HDLc/triglyceride of patients

	Preterm group *N:*53	Term group *N:*99	*p*
Monocytes (10^6^/mL)	0.43 ± 0.13	0.39 ± 0.11	0.088
HDLc (mg/dl)	46 ± 7.3	53 ± 11.6	** < 0.001**
LDLc (mg/dl)	92.4 ± 30.4	93 ± 21.8	**0.021**
Total cholesterol (mg/dl)	158.4 ± 36.8	159.3 ± 29.8	0.178
Triglyceride (mg/dl)	102.9 ± 55.5	94.7 ± 43.8	0.565
MHR	0.009 ± 0.003	0.007 ± 0.003	**0.002**
HDLc/LDLc	0.57 ± 0.31	0.59 ± 0.17	**0.018**
HDLc/total cholesterol	0.3 ± 0.08	0.33 ± 0.07	0.123
HDLc/triglyceride	0.58 ± 0.32	0.69 ± 0.37	0.059

*HDLc* high-density lipoprotein cholesterol, *LDLc* low-density lipoprotein cholesterol, *MHR* monocyte-to-HDLc ratio

Pearson correlation analysis showed that HDLc levels and the HDLc/TC ratio were negatively related to PTD rates (*r* =  − 0.308, *p* ≤ 0.001; *r* =  − 0.186, *p* = 0.021, respectively), whereas a positive association was observed with mean birth week (*r* = 0.243, *p* = 0.003). Spearman correlation analysis revealed a negative correlation between MHR and mean birth week (*r* =  − 0.208, *p* = 0.001) as well as birth weight (*r* =  − 0.204, *p* = 0.001), and a positive correlation with PTD rates (*r* = 0.250, *p* = 0.002).

**Fig. 2:**
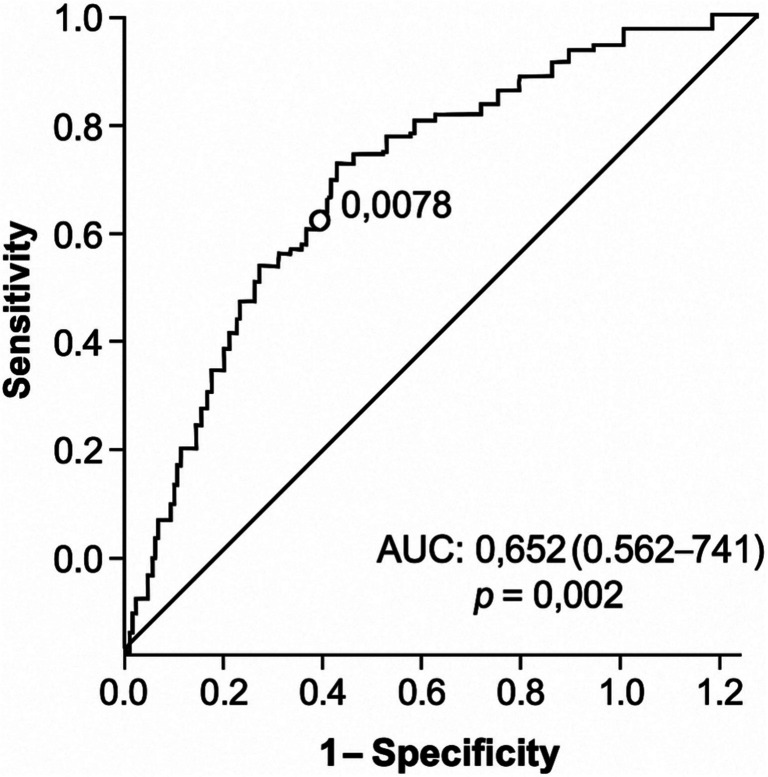
Receiver operating curve for MHR for the predicting preterm delivery. MHR: monocyte-to-high-density lipoprotein ratio

At a cut-off level of 0.0078, MHR accurately predicted occurrence of PTD AUC = 0.652 (95% confidence interval 0.562–0.741, *p* = 0.002) with sensitivity and specificity rates of 64.2% and 58.6%, respectively (Fig. 2).

## Discussion

Preterm delivery remains the leading cause of perinatal mortality and morbidity, contributing to approximately 70% of neonatal deaths and nearly 50% of long-term neurological impairments. Therefore, early identification of high-risk populations and the implementation of effective strategies to prevent preterm labor and its complications are critical priorities in obstetric care [3, 4]. This study examines the relationship between maternal lipid levels MHR during the first trimester of pregnancy and their potential impact on the incidence of preterm birth.

Preterm delivery should be regarded as a syndrome resulting from multiple contributing factors. Pathological mechanisms such as intrauterine infection, intrauterine inflammation, decidual bleeding, ischemia, abnormal allograft reaction, activation of the maternal/fetal hypothalamic–pituitary–adrenal axis, uterine strain, and cervical insufficiency play significant roles in the onset of preterm labor. However, none of these factors provide causal pathways to elucidate PTD [3, 4]. Numerous studies have been conducted to investigate the association between maternal lipid levels and the occurrence of PTD [10–12]. According to the Catov et al., elevated cholesterol and triglyceride levels before or at 15 weeks of gestation have been associated with an increased risk of PTD, particularly in overweight women delivering before 34 weeks. Notably, a reduced triglyceride response in early pregnancy was observed among women delivering before 34 weeks, suggesting a potential metabolic alteration contributing to PTD [12]. A systematic review analyzing the relationship between maternal lipid profiles and spontaneous preterm delivery suggested that high triglyceride levels may be associated with an increased risk, whereas no significant association was found for HDLc and LDLc cholesterol levels. However, the review primarily focused on general lipid alterations throughout pregnancy rather than first-trimester-specific changes [17]. In contrast, our study highlights the significant role of reduced HDLc levels and a lower HDLc/LDLc ratio in the first trimester as potential predictors of PTD. Unlike total cholesterol and triglycerides, which did not show a statistically significant association in our cohort, early gestational reductions in HDLc and an lower HDLc/LDLc ratio may reflect an altered maternal metabolic state contributing to PTD pathogenesis. These findings are supported by prior research, including studies by Kramer et al. and Bartha et al., which reported lower HDLc levels in cases with spontaneous preterm delivery [18, 19]. These observations suggest that HDLc has a protective role in pregnancy, and increased levels may reduce PTD risk. Our study adds to this evidence by demonstrating that first-trimester HDLc and the ratio of HDLc to LDLc are both independent predictors of PTD risk. Further work is required to determine whether early pregnancy interventions that alter HDLc levels can reduce PTD risk and improve perinatal outcomes.

Current evidence suggest that inflammatory markers, like the monocyte-to-HDLc ratio, may be associated with the pathogenesis of adverse pregnancy outcomes, including preeclampsia and gestational diabetes mellitus [14, 16]. MHR is a recently discovered biomarker that reflects systemic inflammation and oxidative stress, and both are directly implicated in pregnancy complications. Melekoglu et al. have demonstrated that elevated MHR levels correlate with endothelial dysfunction and placental insufficiency, mechanisms that can lead to preeclampsia [14]. Previous research has also established that MHR is exceptionally high in new-onset diabetes, metabolic syndrome, and cardiovascular disease [20, 21]. This is also in line with the role of MHR as a marker of systemic inflammation and oxidative stress, processes that are implicated in PTD pathogenesis. Our findings are in agreement with this growing evidence since we observed extremely elevated first-trimester MHR levels in women with PTD compared to women with term pregnancies. This suggests that a heightened inflammatory response during early pregnancy may predispose individuals to preterm labor.

Based on the association of MHR with preterm risk, additional studies would be valuable to pursue the prognostic value of this marker and potential interventions to modulate maternal inflammatory responses during early gestation. Incorporating MHR testing into standard prenatal screening could offer an additional method of identifying high-risk pregnancy and guiding early preventive treatment. To our best knowledge, this is the first study to investigate the correlation between first-trimester MHR levels and preterm delivery. Our study demonstrates that low HDLc levels, reduced HDLc/LDLc ratio, and elevated MHR during early gestation are positively correlated with the risk of preterm delivery, suggesting that dyslipidemia and systemic inflammation both have significant roles in its pathogenesis. Although our findings indicate statistically significant associations between MHR, HDLc/LDLc ratio, and the risk of preterm delivery, the predictive capacity of MHR alone appears to be limited. The ROC analysis yielded an AUC of 0.652, which suggests only modest discriminatory performance. While this value demonstrates a statistically significant relationship, it does not meet conventional thresholds for strong clinical utility. Therefore, MHR may be better interpreted as a supportive biomarker within a broader risk assessment model, rather than a standalone diagnostic tool. Future studies should explore the additive predictive value of MHR in combination with other clinical, biochemical, and imaging parameters to enhance risk stratification for preterm birth.

## Data Availability

The datasets used and/or analyzed during the current study are not publicly available but may be available from the corresponding author upon reasonable request.
